# Exploring the potential of artificial intelligence to enhance the writing of english academic papers by non-native english-speaking medical students - the educational application of ChatGPT

**DOI:** 10.1186/s12909-024-05738-y

**Published:** 2024-07-09

**Authors:** Jiakun Li, Hui Zong, Erman Wu, Rongrong Wu, Zhufeng Peng, Jing Zhao, Lu Yang, Hong Xie, Bairong Shen

**Affiliations:** 1 Department of Urology and Institutes for Systems Genetics, Frontiers Science Center for Disease-related Molecular Network, West China Hospital, Sichuan University, Chengdu, 610041 China; 2 West China Hospital, West China School of Medicine, Sichuan University, No. 37, Guoxue Alley, Chengdu, 610041 China; 3 Institutes for Systems Genetics, Frontiers Science Center for Disease-related Molecular Network, West China Hospital, Sichuan University, Chengdu, 610041 China; 4 Department of Neurosurgery, the First Affiliated Hospital of Xinjiang Medical University, Urumqi, 830054 China

**Keywords:** Large language model, ChatGPT, Medical education, Medical english, Academic writing

## Abstract

**Background:**

Academic paper writing holds significant importance in the education of medical students, and poses a clear challenge for those whose first language is not English. This study aims to investigate the effectiveness of employing large language models, particularly ChatGPT, in improving the English academic writing skills of these students.

**Methods:**

A cohort of 25 third-year medical students from China was recruited. The study consisted of two stages. Firstly, the students were asked to write a mini paper. Secondly, the students were asked to revise the mini paper using ChatGPT within two weeks. The evaluation of the mini papers focused on three key dimensions, including structure, logic, and language. The evaluation method incorporated both manual scoring and AI scoring utilizing the ChatGPT-3.5 and ChatGPT-4 models. Additionally, we employed a questionnaire to gather feedback on students’ experience in using ChatGPT.

**Results:**

After implementing ChatGPT for writing assistance, there was a notable increase in manual scoring by 4.23 points. Similarly, AI scoring based on the ChatGPT-3.5 model showed an increase of 4.82 points, while the ChatGPT-4 model showed an increase of 3.84 points. These results highlight the potential of large language models in supporting academic writing. Statistical analysis revealed no significant difference between manual scoring and ChatGPT-4 scoring, indicating the potential of ChatGPT-4 to assist teachers in the grading process. Feedback from the questionnaire indicated a generally positive response from students, with 92% acknowledging an improvement in the quality of their writing, 84% noting advancements in their language skills, and 76% recognizing the contribution of ChatGPT in supporting academic research.

**Conclusion:**

The study highlighted the efficacy of large language models like ChatGPT in augmenting the English academic writing proficiency of non-native speakers in medical education. Furthermore, it illustrated the potential of these models to make a contribution to the educational evaluation process, particularly in environments where English is not the primary language.

**Supplementary Information:**

The online version contains supplementary material available at 10.1186/s12909-024-05738-y.

## Introduction

Large language models (LLMs) are artificial intelligence (AI) tools that have remarkable ability to understand and generate text [1, 2]. Trained with substantial amounts of textual data, LLMs have demonstrated their capability to perform diverse tasks, such as question answering, machine translation, and writing [3, 4]. In 2022, Open AI released a LLM called ChatGPT [5]. Since its inception, ChatGPT has been widely applied in medicine domain, especially after testing, it can demonstrate the medical level that meets the requirements of passing the United States Medical Licensing Exam [6]. It can provide personalized learning experience according to the preference style of medical students [7]. Research has shown that the explanations provided by ChatGPT are more accurate and comprehensive than the explanations of basic principles provided in some standardized higher education exams [8]. Therefore, many researchers believe that ChatGPT may improve students’ problem-solving ability and reflective learning [9].

Writing English language based academic papers is very important for the development of medical students in universities. China is a non-native English-speaking country with a large population of medical students, so it is necessary to provide medical education and offer relevant courses, especially to cultivate their ability to write English academic papers [10]. This is essential for future engagement in scientific research and clinical work within the field of medicine. However, the ability of these non-native English-speaking medical students in writing English papers is relatively limited, and they need continuous training and improvement [11].

LLMs can be used to generate and modify text content and language styles, and can be applied to the quality improvement of scientific papers [12, 13]. ChatGPT exhibits considerable potential in medical paper writing, assist in literature retrieval, data analysis, knowledge synthesis and other aspects [14]. Students received AI-assisted instruction exhibited improved proficiency in multiple aspects of writing, organization, coherence, grammar, and vocabulary [15]. Additionally, AI mediated instruction can positively impacts English learning achievement and self-regulated learning [16]. LLMs can also perform language translation [13, 17]. Moreover, it can automatically evaluate and score the level of medical writing, and provide modification suggestions for improvement [18]. These studies indicate that incorporating large language models like ChatGPT into medical education holds promise for various advantages. However, their usage must be accompanied by careful and critical evaluation [19]. As far as we know, there is currently no research to evaluate the usability and effectiveness of ChatGPT in medical mini paper writing courses through real classroom teaching scenarios.

Therefore, in this study, we introduce the ChatGPT into real-world medical courses to investigate the effectiveness of employing LLMs in improving the academic writing proficiency for non-native English-speaking medical students. By collecting and analyzing data, we aim to provide evidence of the effectiveness of employing a LLM in improving the English academic writing skills of medical students, thereby facilitating better medical education and improve the scientific research ability and writing skills for students.

## Method

### Participants

The research included 27 third-year medical students from the West China School of Medicine at Sichuan University. These students are all non-native English speakers. These students had concluded their fundamental medical coursework but had not yet embarked on specialized subjects. Exclusion criteria were applied to those who failed to fulfill the requisite homework assignments.

### Materials

Initial Stage: The task involved composing an English academic paper in accordance with the stipulations of English thesis education. Considering the students’ junior academic standing, the composition of a discussion section in paper was not mandated. Each student was tasked with authoring a concise, “mini paper.”

Experimental Phase: Upon the completion of their individual “mini papers,” students had initially submitted these under the label “group without ChatGPT.” Subsequently, they engaged with ChatGPT-3.5 for a period of two weeks to refine their English academic manuscripts. After this period, the revised mini papers were resubmitted under the designation “group with ChatGPT.” Alongside this resubmission, students also provided a questionnaire regarding their experience with ChatGPT. The questionnaire was administered in Mandarin, which is the commonly used language in the research context. We conducted a thorough discussion within our teaching and research group to develop the questionnaire. Two students, who failed to meet the stipulated submission deadline, were excluded from the study.

### Procedures

All mini papers underwent evaluation and scoring based on a standardized scoring criterion. The assessment process encompassed three distinct approaches. Firstly, two teachers independently scored each mini paper using a blind review technique, and the final score was determined by averaging the two assessments. Secondly, scoring was performed using ChatGPT-3.5. Lastly, scoring was conducted using ChatGPT-4.

Evaluation Criteria: The scoring was composed of three dimensions: structure, logic, and language, with each dimension carrying a maximum of 20 points, culminating in a total of 60 points. The scores for each section were categorized into four tiers: 0–5 points (Fail), 6–10 points (Below Average), 11–15 points (Good), and 16–20 points (Excellent). The minimum unit for deduction was 0.5 points.

Structure emphasizes the organization and arrangement of the paper. It ensures that the content is placed in the appropriate sections according to the guidelines commonly found in academic journals. Logic refers to the coherence and progression of ideas within the paper. The logical flow should be evident, with each section building upon the previous ones to provide a cohesive argument. A strong logical framework ensures a systematic and well-supported study. Language refers to the correctness and proficiency of English writing. Proper language expression is essential for effectively conveying ideas and ensuring clear communication, and makes the paper becomes more readable and comprehensible to the intended audience.

Experience questionnaire for ChatGPT: The questionnaire comprised 31 questions, detailed in the attached appendix. (Attachment document)

### Data analysis

The Kruskal-Wallis rank sum test was utilized to assess the baseline scores of students before and after using ChatGPT. A paired t-test was utilized to analyze the impact of ChatGPT on the improvement of students’ assignment quality (manual grading). Univariate regression analysis was conducted to investigate the extent of improvement in assignment quality attributed to ChatGPT. Previous studies have shown discrepancies in language learning and language-related skills between males and females. In order to mitigate any potential biases, we implemented gender correction techniques, which encompassed statistical adjustments to accommodate these gender variations [20–22]. The questionnaire was distributed and collected using the Wenjuanxing platform (Changsha Ran Xing Science and Technology, Shanghai, China. [https://www.wjx.cn]).

Statistical analyses were performed using the R software package (version 4.2.0, The R Foundation, Boston, MA, USA), Graph Pad Prism 9 (GraphPad Software, CA, USA), and Empower (X&Y Solutions Inc., Boston, MA, USA) [23].

## Results

### Manual scoring

Ultimately, the study included 25 participants, with two students being excluded due to late submission of their assignments. These participants were all third-year undergraduate students, including 14 males (56%) and 11 females (44%). The “group without ChatGPT” consisted of 25 participants who wrote mini papers with an average word count of 1410.56 ± 265.32, cited an average of 16.44 ± 8.31 references, and received a manual score of 46.45 ± 3.59. In contrast, the “group with ChatGPT” of 25 participants produced mini papers with an average word count of 1406.52 ± 349.59, cited 16.80 ± 8.10 references on average, and achieved a manual score of 50.68 ± 2.03. Further details are available in Table 1.

**Table 1:** Academic paper scores of the included population before and after using ChatGPT. Data was showed as Mean ± Standard deviation

Grouping	Without ChatGPT	With ChatGPT	*P*-value
Number	25	25	
Manual Structure	15.02 ± 2.16	17.00 ± 0.75	< 0.001
Manual Logic	15.78 ± 1.27	17.00 ± 0.74	< 0.001
Manual Language	15.65 ± 0.71	16.68 ± 0.75	< 0.001
Manual Total Score	46.45 ± 3.59	50.68 ± 2.03	< 0.001
ChatGPT3.5 Structure	14.30 ± 1.45	15.38 ± 2.18	0.01
ChatGPT3.5 Logic	14.06 ± 1.46	15.62 ± 1.34	< 0.001
ChatGPT3.5 Language	13.04 ± 1.81	15.22 ± 1.50	< 0.001
ChatGPT3.5 Total Score	41.40 ± 4.38	46.22 ± 4.05	< 0.001
ChatGPT4 Structure	16.26 ± 2.45	17.66 ± 0.85	0.003
ChatGPT4 Logic	15.84 ± 2.62	17.26 ± 0.88	0.02
ChatGPT4 Language	15.74 ± 2.96	16.76 ± 0.48	0.23
ChatGPT4 Total Score	47.84 ± 7.47	51.68 ± 1.74	0.002
Word count	1410.56 ± 265.32	1406.52 ± 349.59	0.95
References	16.44 ± 8.31	16.80 ± 8.10	0.79

**Fig. 1:**
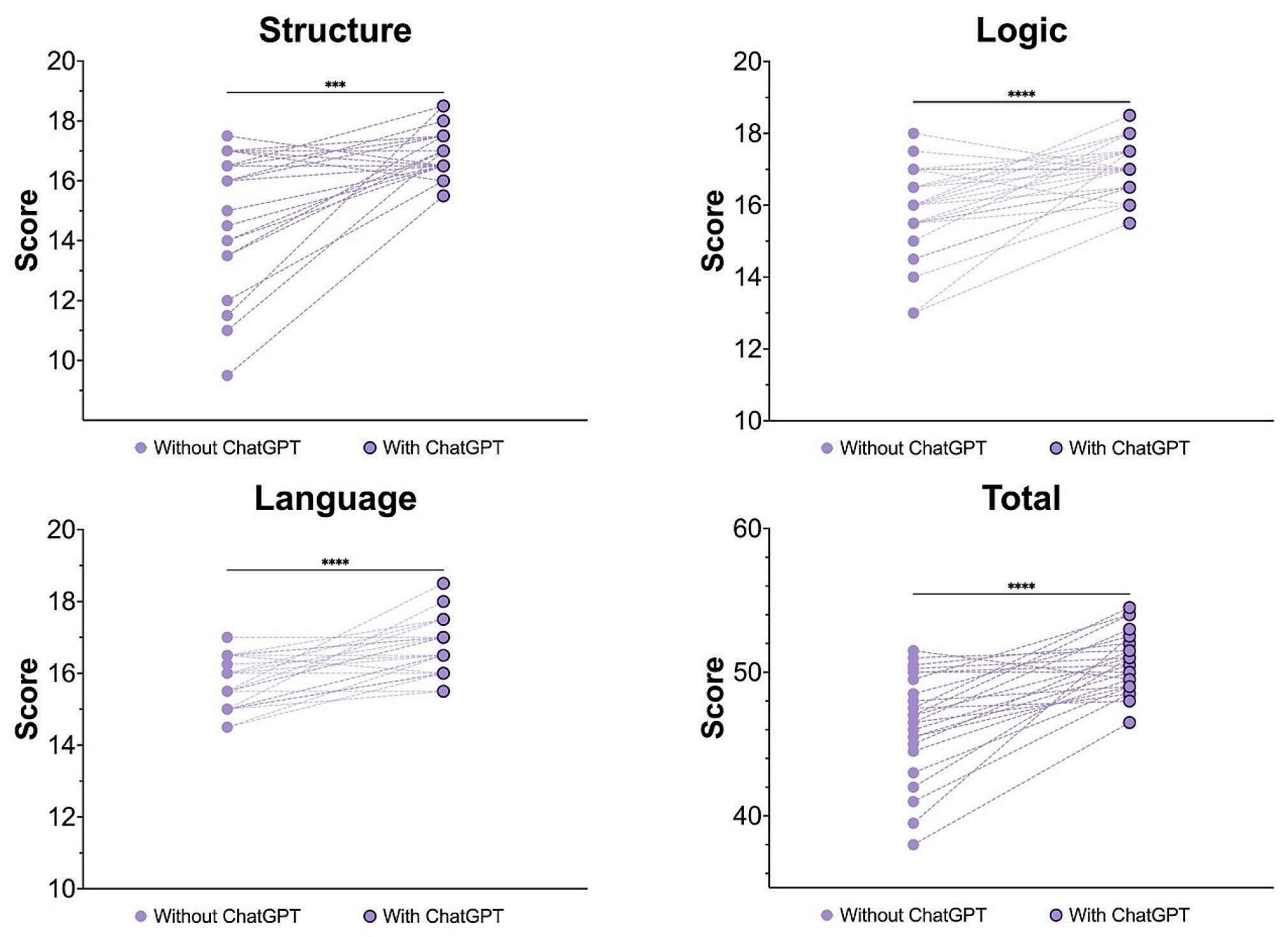
Using ChatGPT improved the quality of students’ academic papers. A statistical analysis of the manual scoring showed that the quality of students’ academic papers improved after using ChatGPT for revision in terms of structure, logic, language, and overall score. The results showed statistical significance. ***p < 0.001, ****p < 0.0001

In terms of manual scoring, medical students demonstrated a significant improvement in the quality of their assignments in the dimensions of logic, structure, language, and overall score after using ChatGPT, as depicted in Fig. 1.

We also conducted a univariate analysis on the impact of ChatGPT on medical students’ academic papers writing across all scoring methods. The results indicated significant improvement in all manual scores and those evaluated by ChatGPT-3.5 for paper structure, logic, language, and total score (all *p* < 0.05). Papers assessed by ChatGPT-4 also showed significant improvements in structure, logic, and total score (all *p* < 0.05). Although the language scores of papers evaluated by ChatGPT-4 did not show a significant difference, a trend of improvement was observed (β 1.02, 95% confidence interval (CI) -0.15, 2.19, *p* = 0.1). After adjusting for gender, multivariate regression analysis yielded similar results, with significant improvements in all dimensions of scoring across all methods, except for the language scores evaluated by ChatGPT-4. The total manual scoring of students’ papers improved by 4.23 (95% CI 2.64, 5.82) after revisions with ChatGPT, ChatGPT-3.5 scores increased by 4.82 (95% CI 2.47, 7.17), and ChatGPT-4 scores by 3.84 (95% CI 0.83, 6.85). Further details are presented in Table 2.

**Table 2:** Comparison of academic paper scores before and after using ChatGPT among the included population. Effect value was reprented as β. Multiple regression analysis adjusted for gender. CI: confidence interval

	Univariate regression analysis		multivariate regression analysis	
	β (95% CI)	*P*-value	β (95% CI)	*P*-value
Manual Structure	1.98 (1.08, 2.88)	< 0.001	1.98 (1.08, 2.88)	< 0.001
Manual Logic	1.22 (0.65, 1.79)	< 0.001	1.22 (0.66, 1.78)	< 0.001
Manual Language	1.03 (0.63, 1.43)	< 0.001	1.03 (0.63, 1.43)	< 0.001
Manual Total Score	4.23 (2.61, 5.85)	< 0.001	4.23 (2.64, 5.82)	< 0.001
ChatGPT3.5 Structure	1.08 (0.05, 2.11)	0.04	1.08 (0.04, 2.12)	0.047
ChatGPT3.5 Logic	1.56 (0.78, 2.34)	< 0.001	1.56 (0.78, 2.34)	< 0.001
ChatGPT3.5 Language	2.18 (1.26, 3.10)	< 0.001	2.18 (1.26, 3.10)	< 0.001
ChatGPT3.5 Total Score	4.82 (2.48, 7.16)	< 0.001	4.82 (2.47, 7.17)	< 0.001
ChatGPT4 Structure	1.40 (0.38, 2.42)	0.01	1.40 (0.38, 2.42)	0.01
ChatGPT4 Logic	1.42 (0.34, 2.50)	0.01	1.42 (0.33, 2.51)	0.01
ChatGPT4 Language	1.02 (-0.15, 2.19)	0.10	1.02 (-0.16, 2.20)	0.10
ChatGPT4 Total Score	3.84 (0.83, 6.85)	0.02	3.84 (0.83, 6.85)	0.02
Word count	-4.04 (-176.08, 168.00)	0.96	-4.04 (-177.27, 169.19)	0.96
References	0.36 (-4.19, 4.91)	0.88	0.36 (-3.97, 4.69)	0.87

### The potential of ChatGPT in scoring support

**Fig. 2:**
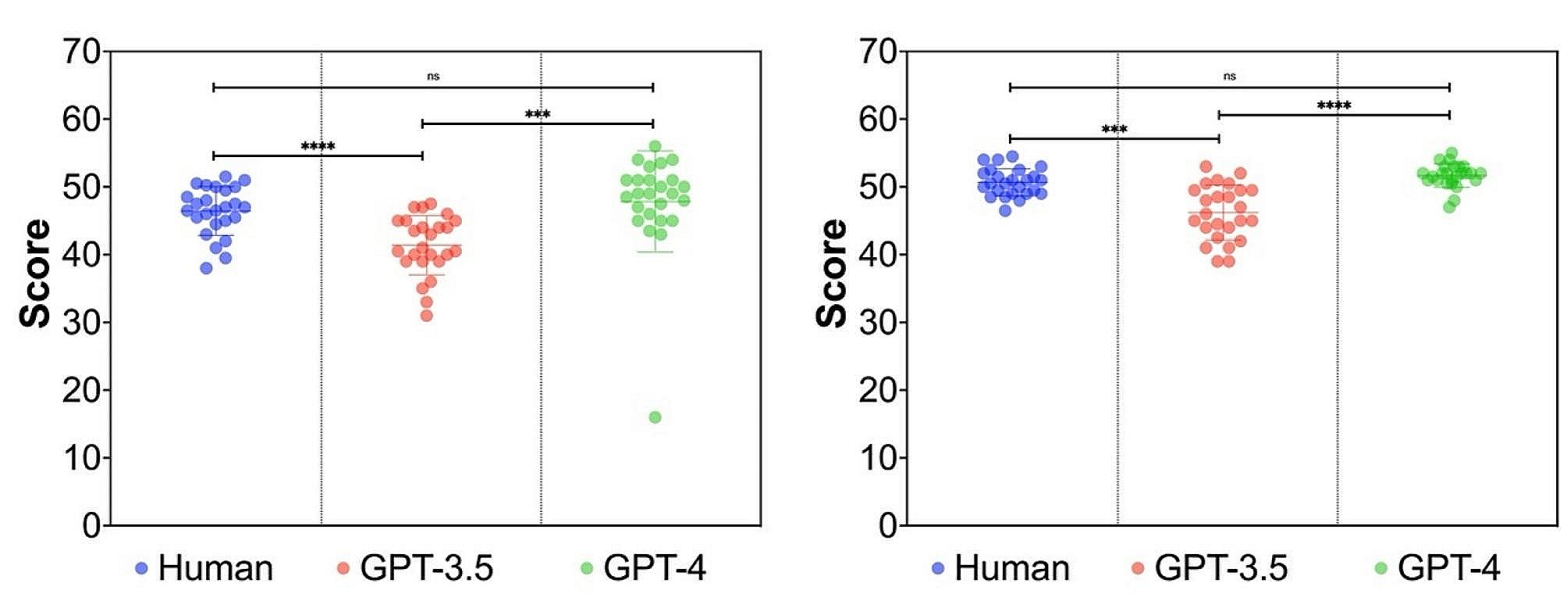
Potential of ChatGPT assisting teachers in evaluating papers. The results showed that there was a significant statistical difference between the scoring results of the GPT3.5 and the manual scoring results, both for the unrevised mini papers (left) and the revised mini papers (right) using ChatGPT. However, there was no significant statistical difference between the scoring results of GPT4 and the manual scoring results, which mean that GPT4 might be able to replace teachers in scoring in the future. ns: no significance, *** *p* < 0.001, **** *p* < 0.0001

Additionally, we investigated whether ChatGPT could assist teachers in assignment assessment. The results showed significant differences between the scores given by the ChatGPT-3.5 and manual grading, both for groups with and without ChatGPT. Interestingly, the scores from ChatGPT-4 were not significantly different from human grading, which suggests that ChatGPT-4 may have the potential to assist teachers in reviewing and grading student assignments (Fig. 2).

### Experience questionnaire

Among the 25 valid questionnaires, social media emerged as the primary channel through which participants became aware of ChatGPT, accounting for 84% of responses. This was followed by recommendations from acquaintances and requirements from schools/offices, each selected by 48% of participants. News media accounted for 44%. (Attachment document)

Regarding the purpose of using ChatGPT (multiple responses allowed), 92% used it mainly to enhance homework quality and improve writing efficiency. 68% utilized ChatGPT for knowledge gathering. 56% employed ChatGPT primarily to improve their language skills. (Attachment document)

In the course of the study, the most widely used feature of ChatGPT in assisting with academic paper writing was English polishing, chosen by 100% of the students, indicating its widespread use for improving the language quality of their papers. Generating outlines and format editing were also popular choices, with 64% and 60% using these features, respectively. (Attachment document)

When asked what they would use ChatGPT for, 92% of participants considered it as a language learning tool for real-time translation and grammar correction. 84% viewed ChatGPT as a tool for assisting in paper writing, providing literature materials and writing suggestions. 76% saw ChatGPT as a valuable tool for academic research and literature review. 48% believed that ChatGPT could serve as a virtual tutor, providing personalized learning advice and guidance. (Attachment document)

Regarding attitudes towards the role of ChatGPT in medical education, 24% of participants had an optimistic view, actively embracing its role, while 52% had a generally positive attitude, and 24% held a neutral stance. This indicates that most participants viewed the role of ChatGPT in medical education positively, with only a minority being pessimistic. (Attachment document)

Among the participants, when asked about the limitations of ChatGPT in medical education, 96% acknowledged the challenge in verifying the authenticity of information; 72% noted a lack of human-like creative thinking; 52% pointed out the absence of clinical practice insights; and 40% identified language and cultural differences as potential issues. (Attachment document)

## Discussion

The results from the participants’ two-week unrestricted usage of the AI model ChatGPT to enhance their assignments indicated a noticeable improvement in the quality of student papers. This suggests that large language models could serve as assistive tools in medical education by potentially improving the English writing skills of medical students. Furthermore, the results of comparative analysis revealed that the ChatGPT-4 model’s evaluations showed no statistical difference from teacher’s manual grading. Therefore, AI might have prospective applications in certain aspects of teaching, such as grading assessments, providing significant assistance to manual efforts.

The results of questionnaire indicate ChatGPT can serve as an important educational tool, beneficial in a range of teaching contexts, including online classroom Q&A assistant, virtual tutor and facilitating language learning [24]. ChatGPT’s expansive knowledge base and advanced natural language processing capability enable it to effectively answer students’ inquiries and offer valuable literature resources and writing advice [25]. For language learning, it offers real-time translation and grammar correction, aiding learners in improving their language skills through evaluation and feedback [26]. ChatGPT can also deliver personalized educational guidance based on individual student needs, enhancing adaptive learning strategies [27]. Furthermore, in this study, the positive feedback of questionnaire for the usage of ChatGPT in English language polishing of academic papers, as well as for generating paper outlines and formatting, underscores its acceptance and recognition among students. The evaluation results of three dimensions reflects a keen focus on enhancing the structural and formatting quality of their papers, demonstrating the large AI language model’s impressive teaching efficacy in undergraduate education.

In the questionnaire assessing ChatGPT’s accuracy and quality, 48% of respondents indicated satisfaction with its performance. However, it’s important to consider that the quality and accuracy of responses from any AI model, including ChatGPT, can be influenced by various factors such as the source of data, model design, and training data quality. These results, while indicative, require deeper research and analysis to fully understand the capabilities and limitations of ChatGPT in this field. Furthermore, ongoing discussions about ethics and data security in AI applications highlight the need for continued vigilance and improvement [28]. Overall, while ChatGPT shows promise in medical education, it is clear that it has limitations that must be addressed to better serve the needs of this specialized field.

Manual grading can be a time-consuming task for teachers, particularly when dealing with a large number of assignments or exams. ChatGPT-4 may provide support to teachers in the grading process, which could free up their time, allowing them to focus on other aspects of teaching, such as providing personalized feedback or engaging with students. However, it may not replace the role of teachers in grading. Teachers possess valuable expertise and contextual knowledge that go beyond simple evaluation of assignments. They consider factors such as student effort, creativity, critical thinking, and the ability to convey ideas effectively. These aspects might be challenging for an AI model to fully capture and evaluate. Furthermore, the use of AI in grading raises important ethical considerations. It is crucial to ensure that the model’s grading criteria align with educational standards and are fair and unbiased.

Despite its potential benefits of using ChatGPT in medical education, it also has limitations, such as language barriers and cultural differences [29, 30]. When inputted with different languages, ChatGPT may have difficulty in understanding and generating accurate responses. Medical terms and concepts vary across different languages, and even slight differences in translation can lead to misunderstandings. Medical education is also influenced by cultural factors. Different cultures have different communication styles, which can impact the way medical information is exchanged. Recognizing and respecting the diversity of cultural perspectives is crucial for providing patient-centered care, and it should be an important part in medical education, which ChatGPT does not excel at. The model may struggle with translating non-English languages, impacting its effectiveness in a global medical education context. Additionally, while ChatGPT can generate a vast amount of text, it lacks the creative thinking and contextual understanding inherent to human cognition, which can be crucial in medical education. Another concern is the authenticity and credibility of the information generated by ChatGPT [31, 32]. In medical education, where accuracy and reliability of knowledge are paramount, the inability to guarantee the truthfulness of the information poses a significant challenge [32–34].

These limitations of ChatGPT in medical education may be addressed and potentially rectified with updates and advancements in AI models. For instance, in this study, the scoring results showed no statistical difference between the ChatGPT-4 model and manual grading, unlike the significant discrepancies observed with the ChatGPT-3.5 model. This suggests that ChatGPT-4 has improved capabilities to assist manual grading by teachers, demonstrating greater intelligence and human-like understanding compared to the ChatGPT-3.5 model. Similar findings have been noted in other research, highlighting the advancements from version 3.5 to 4. For example, there were clear evidences that version 4 achieved better test results than version 3.5 in professional knowledge exams in disciplines such as orthopedics [35], dermatology [36], and ophthalmology [37].

## Conclusion

This study aimed to explore the use of ChatGPT in enhancing English writing skills among non-native English-speaking medical students. The results showed that the quality of students’ writing improved significantly after using ChatGPT, highlighting the potential of large language models in supporting academic writing by enhancing structure, logic, and language skills. Statistical analysis indicated that ChatGPT-4 has the potential to assist teachers in grading. As a pilot study in this field, it may pave the way for further research on the application of AI in medical education. This new approach of incorporating AI into English paper writing education for medical students represents an innovative research perspective. This study not only aligns with the evolving landscape of technology-enhanced learning but also addresses specific needs in medical education, particularly in the context of academic writing. In the future, AI models should be more rationally utilized to further enhance medical education and improve medical students’ research writing skills.

### Electronic supplementary material

Below is the link to the electronic supplementary material.

Supplementary Material 1

## Data Availability

The datasets used and/or analysed during the current study available from the corresponding author on reasonable request.

## Declarations

### AI use in the writing process

During the writing of this work the author(s) used generative AI and/or AI-assisted technologies for the purpose of English language polishing. The author(s) take responsibility for the content and intended meaning of this article.
