# Supplementary material for: Exploring the potential of artificial intelligence to enhance the writing of english academic papers by non-native english-speaking medical students - the educational application of ChatGPT
Source: BMC Med Educ. 2024 Jul 9;24:736. doi: 10.1186/s12909-024-05738-y (PMC11232216; doi:10.1186/s12909-024-05738-y)
Supplement: Supplementary file 1 — Supplementary Material 1 [file 12909_2024_5738_MOESM1_ESM.docx]

Questionnaire after using ChatGPT to revise medical English papers

1. Gender?

| Option | Number | Proportion |
| --- | --- | --- |
| Male | 14 | 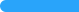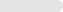56% |
| Female | 11 | 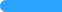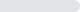44% |

1. How much do you know about ChatGPT before the classroom introduction?

| Option | Number | Proportion |
| --- | --- | --- |
| Never understood | 0 | 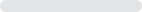0% |
| Basic lack of understanding | 8 | 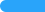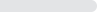32% |
| Neutrality | 12 | 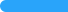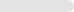48% |
| Familiar | 5 | 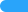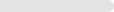20% |
| Very familiar | 0 | 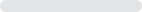0% |

1. How did you learn about ChatGPT? [Multiple choice question]

| Option | Number | Proportion |
| --- | --- | --- |
| Social media | 21 | 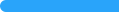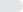84% |
| News media | 11 | 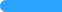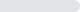44% |
| Recommended by acquaintances | 12 | 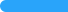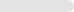48% |
| School/Office Requirements | 12 | 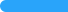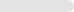48% |
| Other | 0 | 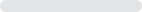0% |
| Never understood | 0 | 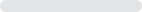0% |

1. Have you ever used intelligent products such as ChatGPT? [Multiple choice question]

| Option | Number | Proportion |
| --- | --- | --- |
| Google Bard, Jasper AI, etc | 2 | 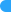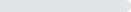8% |
| Siri, Little Love, Tmall Genie, ERNIE Bot, etc | 17 | 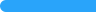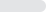68% |
| Other AI intelligent programs | 5 | 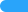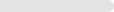20% |
| None | 7 | 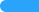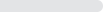28% |

1. What is the main purpose of using ChatGPT? [Multiple choice question]

| Option | Number | Proportion |
| --- | --- | --- |
| Improve homework quality | 23 | 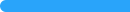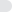92% |
| Improve writing efficiency | 23 | 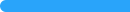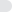92% |
| Improve language expression skills | 14 | 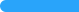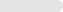56% |
| Conduct knowledge gathering | 17 | 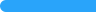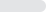68% |
| Other | 1 | 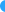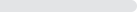4% |

1. In which aspects did you use ChatGPT in this course paper writing assignment? [Multiple choice question]

| Option | Number | Proportion |
| --- | --- | --- |
| Paper English polishing | 25 | 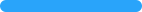100% |
| Generate paper outline | 16 | 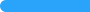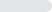64% |
| Generate paper content | 12 | 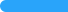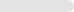48% |
| Generate Text Summary | 10 | 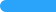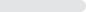40% |
| Generate Paper Title | 6 | 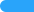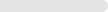24% |
| Paper format modification | 15 | 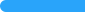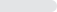60% |
| Calibrate completed papers | 12 | 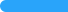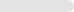48% |
| Generate references | 9 | 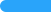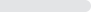36% |
| Provide feedback | 9 | 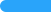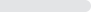36% |
| Other | 0 | 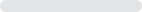0% |

1. In which aspects did you use ChatGPT in writing English emails for this course? [Multiple choice question]

| Option | Number | Proportion |
| --- | --- | --- |
| Content | 18 | 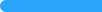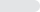72% |
| Structure | 13 | 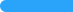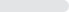52% |
| Mail Format | 15 | 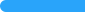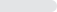60% |
| Language and tone | 15 | 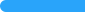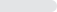60% |
| Strategy and Purpose | 6 | 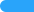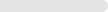24% |
| Format and layout | 11 | 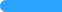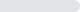44% |
| Attachments and Links | 4 | 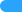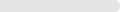16% |
| Other | 0 | 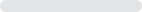0% |

1. How often have you used ChatGPT in the past two weeks?

| Option | Number | Proportion |
| --- | --- | --- |
| Never used before | 0 | 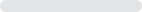0% |
| Only used 1-2 times | 9 | 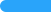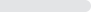36% |
| Use at least once a day on average | 12 | 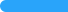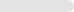48% |
| Use at least 5 times per day on average | 3 | 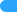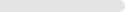12% |
| Always | 1 | 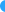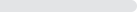4% |

1. How do you think the quality of homework has improved after using ChatGPT?

| Option | Number | Proportion |
| --- | --- | --- |
| 1 No improvement | 0 | 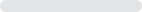0% |
| 2 | 2 | 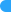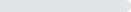8% |
| 3 | 6 | 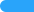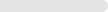24% |
| 4 | 9 | 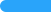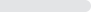36% |
| 5 Significant improvement | 8 | 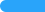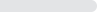32% |

1. What are you interested in about ChatGPT? [Multiple choice question]

| Option | Number | Proportion |
| --- | --- | --- |
| Strong language comprehension ability | 17 | 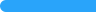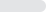68% |
| Suitable for multiple scenarios | 16 | 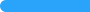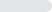64% |
| Efficient | 19 | 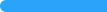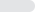76% |
| Reduce workload | 20 | 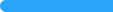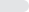80% |
| Strong scalability | 15 | 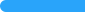60% |
| Strong scientific nature | 10 | 40% |
| Other | 0 | 0% |

1. I think ChatGPT has high effectiveness in education and learning.

| Option | Number | Proportion |
| --- | --- | --- |
| 1 Strongly disagree | 0 | 0% |
| 2 Disagree | 1 | 4% |
| 3 Neutrality | 8 | 32% |
| 4 Agree | 8 | 32% |
| 5 Strongly agree | 8 | 32% |

1. What do you think is the role of ChatGPT in medical education?

| Option | Number | Proportion |
| --- | --- | --- |
| No use | 0 | 0% |
| Not very useful | 1 | 4% |
| Neutrality | 10 | 40% |
| Useful | 6 | 24% |
| Very useful | 8 | 32% |

1. What is your attitude towards the role of ChatGPT in medical education?

| Option | Number | Proportion |
| --- | --- | --- |
| 1 Hold a pessimistic attitude and do not want to use it | 0 | 0% |
| 2 | 0 | 0% |
| 3 Neutrality | 6 | 24% |
| 4 | 13 | 52% |
| 5 Maintain an optimistic attitude and actively use it | 6 | 24% |

1. I am satisfied with the accuracy and quality of ChatGPT's answers.

| Option | Number | Proportion |
| --- | --- | --- |
| 1 Strongly disagree | 0 | 0% |
| 2 Disagree | 4 | 16% |
| 3 Neutrality | 9 | 36% |
| 4 Agree | 7 | 28% |
| 5 Strongly agree | 5 | 20% |

1. After using ChatGPT, it is easier for me to understand and apply the knowledge I have learned.

| Option | Number | Proportion |
| --- | --- | --- |
| 1 Strongly disagree | 0 | 0% |
| 2 Disagree | 1 | 4% |
| 3 Neutrality | 10 | 40% |
| 4 Agree | 7 | 28% |
| 5 Strongly agree | 7 | 28% |

1. ChatGPT has improved my learning efficiency.

| Option | Number | Proportion |
| --- | --- | --- |
| 1 Strongly disagree | 0 | 0% |
| 2 Disagree | 2 | 8% |
| 3 Neutrality | 7 | 28% |
| 4 Agree | 9 | 36% |
| 5 Strongly agree | 7 | 28% |

1. ChatGPT has stimulated my willingness to actively learn.

| Option | Number | Proportion |
| --- | --- | --- |
| 1 Strongly disagree | 0 | 0% |
| 2 Disagree | 2 | 8% |
| 3 Neutrality | 11 | 44% |
| 4 Agree | 6 | 24% |
| 5 Strongly agree | 6 | 24% |

1. After using ChatGPT, my learning motivation has improved.

| Option | Number | Proportion |
| --- | --- | --- |
| 1 Strongly disagree | 0 | 0% |
| 2 Disagree | 3 | 12% |
| 3 Neutrality | 10 | 40% |
| 4 Agree | 6 | 24% |
| 5 Strongly agree | 6 | 24% |

1. ChatGPT provided useful learning resources and guidance.

| Option | Number | Proportion |
| --- | --- | --- |
| 1 Strongly disagree | 0 | 0% |
| 2 Disagree | 1 | 4% |
| 3 Neutrality | 10 | 40% |
| 4 Agree | 8 | 32% |
| 5 Strongly agree | 6 | 24% |

1. After using ChatGPT, I am able to better solve problems and confusions in my studies.

| Option | Number | Proportion |
| --- | --- | --- |
| 1 Strongly disagree | 0 | 0% |
| 2 Disagree | 0 | 0% |
| 3 Neutrality | 9 | 36% |
| 4 Agree | 9 | 36% |
| 5 Strongly agree | 7 | 28% |

1. The use of ChatGPT enabled me to learn more independently and autonomously.

| Option | Number | Proportion |
| --- | --- | --- |
| 1 Strongly disagree | 0 | 0% |
| 2 Disagree | 2 | 8% |
| 3 Neutrality | 11 | 44% |
| 4 Agree | 6 | 24% |
| 5 Strongly agree | 6 | 24% |

1. ChatGPT made my learning process more interactive and personalized.

| Option | Number | Proportion |
| --- | --- | --- |
| 1 Strongly disagree | 0 | 0% |
| 2 Disagree | 3 | 12% |
| 3 Neutrality | 9 | 36% |
| 4 Agree | 6 | 24% |
| 5 Strongly agree | 7 | 28% |

1. The application of ChatGPT has enhanced my learning confidence.

| Option | Number | Proportion |
| --- | --- | --- |
| 1 Strongly disagree | 0 | 0% |
| 2 Disagree | 3 | 12% |
| 3 Neutrality | 11 | 44% |
| 4 Agree | 5 | 20% |
| 5 Strongly agree | 6 | 24% |

1. ChatGPT has increased my enjoyment and satisfaction in learning.

| Option | Number | Proportion |
| --- | --- | --- |
| 1 Strongly disagree | 0 | 0% |
| 2 Disagree | 0 | 0% |
| 3 Neutrality | 11 | 44% |
| 4 Agree | 9 | 36% |
| 5 Strongly agree | 5 | 20% |

1. Will you continue to use ChatGPT to help you with your learning in the future?

| Option | Number | Proportion |
| --- | --- | --- |
| 1 Never | 0 | 0% |
| 2 | 0 | 0% |
| 3 | 3 | 12% |
| 4 | 13 | 52% |
| 5 Very willing | 9 | 36% |

1. Would you recommend ChatGPT to your friends and classmates around you?

| Option | Number | Proportion |
| --- | --- | --- |
| 1 Strongly not recommended | 0 | 0% |
| 2 | 0 | 0% |
| 3 | 6 | 24% |
| 4 | 12 | 48% |
| 5 Highly Recommended | 7 | 28% |

1. Do you think ChatGPT can replace traditional writing teaching?

| Option | Number | Proportion |
| --- | --- | --- |
| 1 Irreplaceable | 2 | 8% |
| 2 | 6 | 24% |
| 3 | 11 | 44% |
| 4 | 4 | 16% |
| 5 Complete substitution | 2 | 8% |

1. What do you think ChatGPT can meet? [Multiple choice question]

| Option | Number | Proportion |
| --- | --- | --- |
| Academic tutoring, such as papers, code self checks, etc | 18 | 72% |
| Resource integration, such as information collection, integration, processing, etc | 18 | 72% |
| Life services, such as chatting, auxiliary service decision-making, etc | 16 | 64% |
| Creation in literary and media related fields, such as creating music, point data, stories, etc | 9 | 36% |
| Language processing, such as translation, email writing, copywriting, etc | 20 | 80% |
| Program programming, such as basic programming work, code output, etc | 11 | 44% |
| Attempting out of interest without purpose | 11 | 44% |
| Other | 0 | 0% |

1. What would you do with ChatGPT? [Multiple choice question]

| Option | Number | Proportion |
| --- | --- | --- |
| ChatGPT serves as a Q&A assistant in online classrooms, used to answer students' questions and solve doubts. | 19 | 76% |
| ChatGPT serves as an auxiliary tool for paper writing, providing literature and writing suggestions. | 21 | 84% |
| ChatGPT is a language learning aid tool used to provide real-time translation and grammar correction. | 23 | 92% |
| ChatGPT serves as a virtual mentor to provide personalized learning advice and guidance to students. | 12 | 48% |
| Other | 0 | 0% |

1. What are the limitations of ChatGPT's application in medical education in your opinion? [Multiple choice question]

| Option | Number | Proportion |
| --- | --- | --- |
| Lack of clinical practice | 13 | 52% |
| Language and cultural differences | 10 | 40% |
| Lack of creativity in human thinking | 18 | 72% |
| Unable to determine the authenticity of the information | 24 | 96% |
| Other | 0 | 0% |
